# Does flower phenology mirror the slowdown of global warming?

**DOI:** 10.1002/ece3.1503

**Published:** 2015-05-15

**Authors:** Susanne Jochner, Annette Menzel

**Affiliations:** 1 Physical Geography/Landscape Ecology and Sustainable Ecosystem Development, Catholic University Eichstätt-Ingolstadt, Ostenstraβe 1885072, Eichstätt, Germany; 2 Ecoclimatology, Department of Ecology and Ecosystem Management, Technische Universität München, Hans-Carl-von-Carlowitz-Platz 285354, Freising, Germany; 3 Institute for Advanced Study, Technische Universität MünchenLichtenbergstraße 2 a, 85748, Garching, Germany

**Keywords:** Bayesian statistics, climate change, flowering, multiple change-point model, phenology, trend

## Abstract

Although recent global warming trends in air temperature are not as pronounced as those observed only one decade ago, global mean temperature is still at a very high level. Does plant phenology – which is believed to be a suitable indicator of climate change – respond in a similar way, that is, does it still mirror recent temperature variations? We explored in detail long-term flowering onset dates of snowdrop, cherry, and lime tree and relevant spring temperatures at three sites in Germany (1901–2012) using the Bayesian multiple change-point approach. We investigated whether mean spring temperature changes were amplified or slowed down in the past decade and how plant phenology responded to the most recent temperature changes. Incorporating records with different end points (i.e., 2002 and 2012), we compared differences in trends and inferred possible differences caused by extrapolating phenological and meteorological data. The new multiple-change point approach is characterized by an enhanced structure and greater flexibility compared to the one change point model. However, the highest model probabilities for phenological (meteorological) records were still obtained for the one change point (linear) model. Marked warming trends in the recent decade were only revealed for mean temperatures of March to May, here better described with one or two change point models. In the majority of cases analyzed, changes in temperatures were well mirrored by phenological changes. However, temperatures in March to May were linked to less strongly advancing onset dates for lime tree flowering during the period 1901-2012, pointing to the likely influence of photoperiodic constraints or unfulfilled chilling requirements. Due to the slowdown of temperature increase, analyses conducted on records ending in 2002 demonstrated distinct differences when compared with records ending in 2012. Extrapolation of trends could therefore (along with the choice of the statistical method) lead to distinctly different results and most recent data should be integrated in order not to over- or underestimate future phenological changes.

## Introduction

Observed temperature data confirm that climatic change is unequivocal: During the period 1880–2012, the mean global air temperature increased by 0.85°C (IPCC 2013). The warming trend was especially strong in the last four decades (Luterbacher et al. 2004; Hansen et al. 2006), and several years of the last two decades were among the warmest since the beginning of instrumental observations 160 years ago (Hansen et al. 2010; Jones et al. 2011; IPCC 2013). The most recent three decades in Germany, for example, included 24 years that were warmer than the mean of the reference period 1961–1990 (DWD 2013).

Conversely, in recent years, global temperature did not continue to increase at a similar magnitude: Recent analyses showed that the 5-year running mean global temperature has been flat for a decade (Hansen et al. 2013). Simulated global warming of the Coupled Model Intercomparison Project Phase 5 (CMIP5) for the past 20 years (1993–2012, + 0.30°C/decade) overestimated recent observations (+ 0.15°C); however, the possible causes for this inconsistency – ranging from errors in external forcing, model response, and internal climate variability – are not yet fully understood (Fyfe et al. 2013). Nevertheless, it is important to note that global temperature is at a much higher level now than decades ago and is also expected to rise further (Hansen et al. 2013; IPCC 2013). Globally, the year 2014 even ranks as the warmest year (14.59°C, +0.69°C) since the beginning of instrumental observations in 1880 (NOAA 2014).

Plant phenology is a useful tool in climate change research as phenological spring events such as flowering or leaf unfolding are particularly sensitive to temperature (Sparks and Carey 1995; Menzel and Fabian 1999). Therefore, phenology has frequently been used to detect the impacts of climate change on plants (Parmesan and Yohe 2003; Root et al. 2003; Menzel et al. 2006; Rosenzweig et al. 2008). Advancing trends in phenological onset dates have been reported for a huge number of phases, both in plants and animals, and for many regions worldwide. Global meta-analyses documented a mean advance of spring phenophases by 2.3 and 5.1 days per decade, respectively (Parmesan and Yohe 2003; Root et al. 2003). However, recently also nonsignificant linear trends or delays in onset dates were reported (Cook et al. 2012; Luedeling et al. 2013; Pope et al. 2013). There exists a number of recent studies reporting nonlinear relationships between phenology and temperature (Sparks et al. 2011; Newnham et al. 2013), but also further linear phenological advances under recent record-breaking spring temperatures (Ellwood et al. 2013). Thus, it remains uncertain whether plant phenology is able or not to keep pace with climate change.

Factors discussed responsible for a weakened response are, for example, photoperiodic constraints and unfulfilled chilling requirements (Körner and Basler 2010; Laube et al. 2014). The latter seems to be of considerable importance in a changing climate as the amount of winter chilling has to be sufficient to break dormancy. Although this requirement is species-specific (Hänninen and Tanino 2011), very warm winter months may result in delayed phenological onset dates – irrespective of the temperature increase in spring (Murray et al. 1989; Partanen et al. 1998; Heide 2003; Dantec et al. 2013; Luedeling et al. 2013).

In light of recent slowdown of temperature increase, valuable perceptions can be obtained by comparing former results that predicted strong advances in plant phenology with projections based on additional information of recent observational data. In prior publications (Dose and Menzel 2004, 2006), the Bayesian approach of nonparametric function estimation was applied to long-term flowering data of *Galanthus nivalis* L. (snowdrop), *Prunus avium* L. (sweet cherry), and *Tilia platyphyllos* Scop. (lime tree) in Geisenheim (Germany) for the period 1896–2002. This approach included, in the model comparison besides constant and linear change, a one change point model (see also Schleip et al. 2006, 2009), which was recently further developed to a multiple change-point approach as presented in Henneken et al. (2013), Pope et al. (2013), and Menzel et al. (2015).

We now applied the multiple change-point approach to the phenological time series used in Dose and Menzel (2004) enhanced by data of the last 10 years (up to 2012), and of two further sites and of temperature. The major aims of this study were as follows:

- To investigate the enhanced structure of the multiple change-point approach.
- To conduct a reanalysis for the time series 1901–2002 (Dose and Menzel 2004) in order to assess the predictive power of extrapolating phenological and temperature trends in comparison with the rates of change obtained from the longer time series (1901–2012).
- To analyze how recent climate change and its slowdown of temperature increase, also referred as “hiatus”, “pausing,” or “standstill,” is mirrored in our phenological data.

## Materials and Methods

### Phenological data

**Figure 1:**
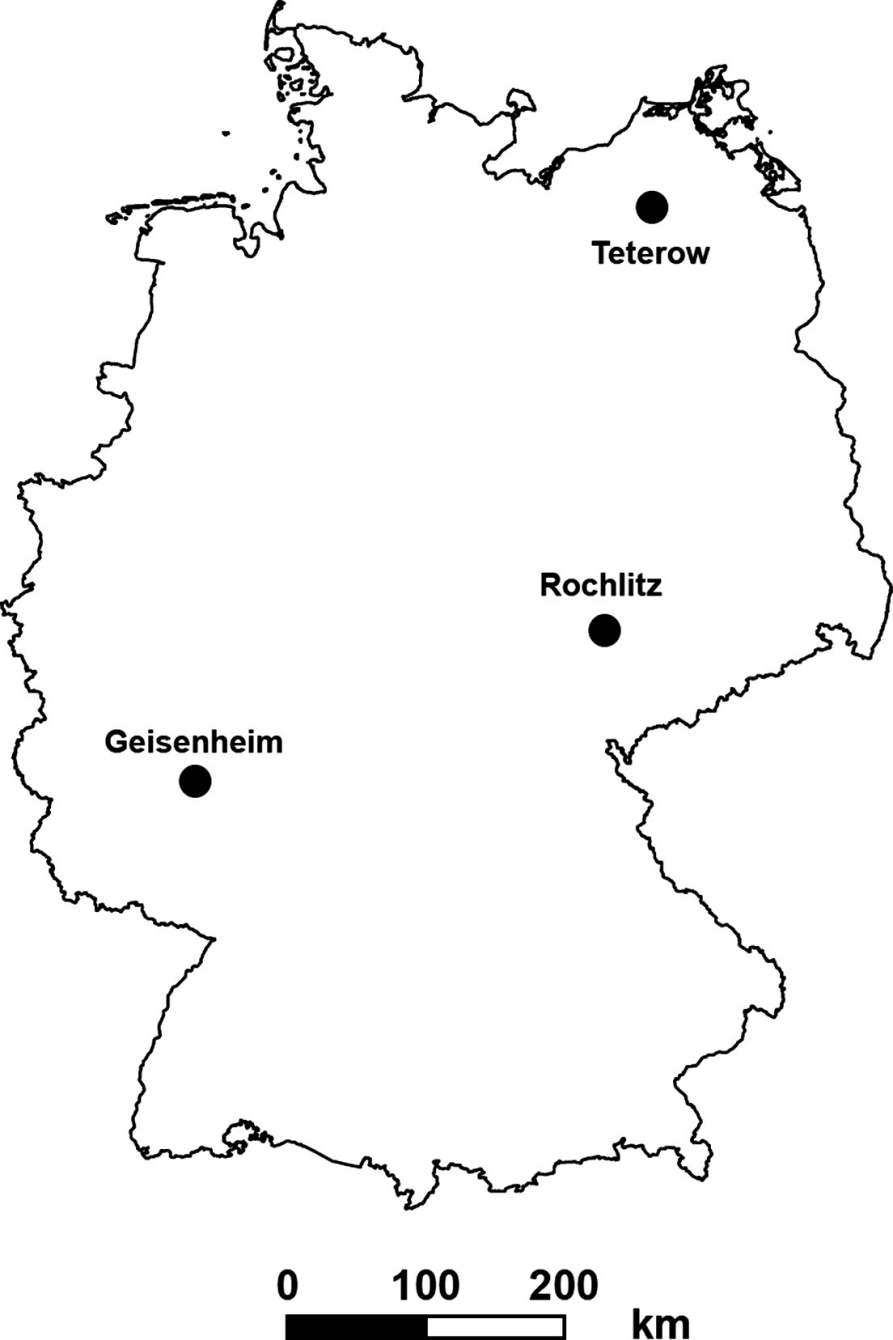
Location of the three selected phenological DWD stations Geisenheim, Rochlitz, and Teterow in Germany.

The selected phenophases were identical with those analyzed by Dose and Menzel (2004): flowering onset of snowdrop, cherry, and lime tree. Besides Geisenheim, we additionally incorporated long-term data (1901–2012) from the stations at Rochlitz and Teterow (see Table1 and Fig.1). Flowering of snowdrop is defined as the date when the exterior pedals are open and their stamen in the interior pedals gets visible. Cherry flowering occurs when the first flowers are completely open and their yellow stamen is visible. Flowering of lime tree is defined as the date when the first flowers of their cymes are completely open and a strong scent is emitted (DWD 1991).

**Table 1:** Selected phenological sites, their geographical information, and number of observations for snowdrop, cherry, and lime tree flowering

Stations	Latitude [°N]	Longitude [°O]	Altitude [m]	Number of observations (1901–2012)		
				Snowdrop	Cherry	Lime tree
Geisenheim	49.99	7.95	110	106	101	106
Rochlitz	51.03	12.80	180	110	101	106
Teterow	53.78	12.58	20	108	101	106

We used the long-term phenological records compiled by Menzel and Testka (2002) and Menzel et al. (2005) from different sources and networks: from the historical phenological database of the German Meteorological Service (DWD; Ihne –1941^1883^), Schnelle and Witterstein (1952), meteorological yearbooks (DWD 1948–1952^1949^, 1951^1950^, ^1960^), Seyfert (, 1960, 1961^1957^), the Meteorological and Hydrological Service of the former East Germany (1961–1990), and the current phenological network of the DWD. Recent data were also obtained from this latter network.

Phenological data were analyzed for two different time periods: 1901–2002 in order to compare the results with the study of Dose and Menzel (2004) and 1901–2012 which represents the most recent available data.

### Temperature data

Temperature data were obtained from the DWD. As station data hardly extend back to the beginning of the 20th century, we used 1-km-gridded monthly means of air temperature which are available for the period 1901 onwards. Mean temperatures of January to March were linked to flowering onset dates of snowdrop, means of February to April to cherry flowering, and means of March to May to lime tree flowering. These mean temperatures were shown to maximize correlations with the respective phenological onset dates (analyses not shown, see Dose and Menzel 2004, 2006).

### Methods

We analyzed all observational time series in terms of a constant, a linear, and a multiple change-point model (see Henneken et al. 2013; Pope et al. 2013; Menzel et al. 2015). Whereas the previously used one change point model for a time series of N entries (Dose and Menzel 2004, 2006) employed N – 2 possible functions consisting of two linear segments and matching at the change point (cpt), the multiple change-point model constitutes its generalization, allowing for polygons with an arbitrary number of change points n_cpt_. Consequently, this function is a piecewise linear continuous function consisting of n_cpt_+1 linear segments matching at the change points. Instead of conventional least squares fitting, we applied Bayesian nonparametric function estimation to these models. Consequently, not only the least square result, a triangle with peak at the change point in case of the one change point model and in the multiple case, a polygon with several change points, but also neighboring, less optimal configurations are included in the data fitting of the Bayesian approach. This means that not only the optimum change point configuration is considered, but also all possible configurations including the respective probabilities of the change point configurations. Our function fit becomes then a superposition of the polygons for all configurations weighted with their respective probabilities. However, for n_cpt_ change points, the number of possible configurations scales approximately as N^ncpt^. As their deterministic evaluation becomes computationally expensive, we used Monte Carlo (MC) approximations to the sums. Thus, for all multiple change-point solutions, Monte Carlo evaluations have been implemented in terms that the results of the N_MC_ change point configurations weighted by their respective probabilities are summed up and divided by N_MC_. The resulting function is no longer a polygon and may show, depending on the number of change points, a considerable flexibility. Within the Bayesian probability theory, it is also possible to determine the probability of the number of change points. However, according to the Ockham's razor (Garret 1991), the maximum of the probability is given by the trade-off between better fit with increasing number of change points and the associated increasing complexity of the data description.

At this stage of the calculation, we obtained constant, linear, and change point fits of the data each with a different probability attached to it. As our data did not fit best with a higher number of change points, the analyses were restricted to a maximum of two change points (see Table2). Our final representation of the data is an average over these fits weighted with their respective probability, thus eliminating the final parameter n_cpt_. We calculated the estimates of the fit function and their associated uncertainties. The derivatives of the fit function correspond to the rate of change in days/year for phenological data (positive values: delays, negative: advancements) and in °C/year for temperature data (positive values: increases, negative: decreases).

**Table 2:** Model probabilities for flowering of snowdrop, cherry, and lime tree at Geisenheim, Rochlitz, and Teterow (1901–2012) in respect to a constant, linear, one change point, and two change point model. Bold values indicate the respective highest model probability

Model	Geisenheim			Rochlitz			Teterow		
	Snowdrop	Cherry	Lime tree	Snowdrop	Cherry	Lime tree	Snowdrop	Cherry	Lime tree
Constant	0.000	0.003	0.000	0.050	0.141	0.000	0.004	0.041	0.000
Linear	0.000	0.193	0.005	0.110	0.366	0.000	0.008	0.166	0.000
One change point	**0.534**	**0.701**	**0.824**	**0.503**	**0.454**	**0.887**	**0.857**	**0.649**	**0.822**
Two change point	0.466	0.102	0.171	0.337	0.039	0.113	0.130	0.144	0.178

The mathematical model formulation, derivation of the posterior distribution, and prior specifics (Jeffrey's prior) were adopted from Henneken et al. (2013). Credible intervals were obtained from MCMC (Markov Chain Monte Carlo) sampling with a script run under Delphi 6.0 (Henneken et al. 2013).

The predictive power of our models was assessed using ME (model efficiency) which is defined as ME = (SS_tot_ − SS_res_)/SS_tot_ where SS_tot_ is the sum of the squared deviations of the observations from their mean and SS_res_ the sum of squared residuals of the model fit.

Further details on the methodology of multiple change-point analyses can be found in Henneken et al. (2013), Pope et al. (2013), and Menzel et al. (2015).

## Results

### Bayesian model choice

Analyzing the appropriateness of different phenological models, we found that the one change point model was most suitable for the analyzed data. Table2 shows the probabilities for the model fit of the flowering records of the three species at Geisenheim, Rochlitz, and Teterow as a function of the number of pivots (n_p_ = n_cpt_ + 2). The highest model probabilities (0.454 ≤  *P *≤* *0.887) were obtained when the one change point model was considered. Associated model efficiencies – which are impaired by a temperature-related year-to-year variability – ranged between 0.3 and 25.5%.

In contrast, for temperature data, the linear model revealed the highest model probabilities, in seven of nine cases (0.543 ≤  *P *≤* *0.95, Table3). For March to May temperatures, the one change point model fitted best at Geisenheim and the two change point model at Teterow and model efficiency varied between 1.1 and 19.2%.

**Table 3:** Model probabilities for the selected temperature data (T 1_3: mean of January to March, T 2_4: mean of February to April, T 3_5: mean of March to May) at Geisenheim, Rochlitz, and Teterow in respect to a constant, linear, one change point, and two change point model. Bold values indicate the highest model probability

Model	Geisenheim			Rochlitz			Teterow		
	T 1_3	T 2_4	T 3_5	T 1_3	T 2_4	T 3_5	T 1_3	T 2_4	T 3_5
Constant	0.050	0.010	0.000	0.286	0.209	0.021	0.302	0.246	0.033
Linear	**0.909**	**0.950**	0.260	**0.622**	**0.728**	**0.462**	**0.556**	**0.543**	0.065
One change point	0.037	0.037	**0.586**	0.082	0.058	0.444	0.127	0.181	0.284
Two change point	0.004	0.003	0.154	0.009	0.005	0.072	0.016	0.030	**0.618**

### Model-averaged functions and temporal rates of change

**Figure 2:**
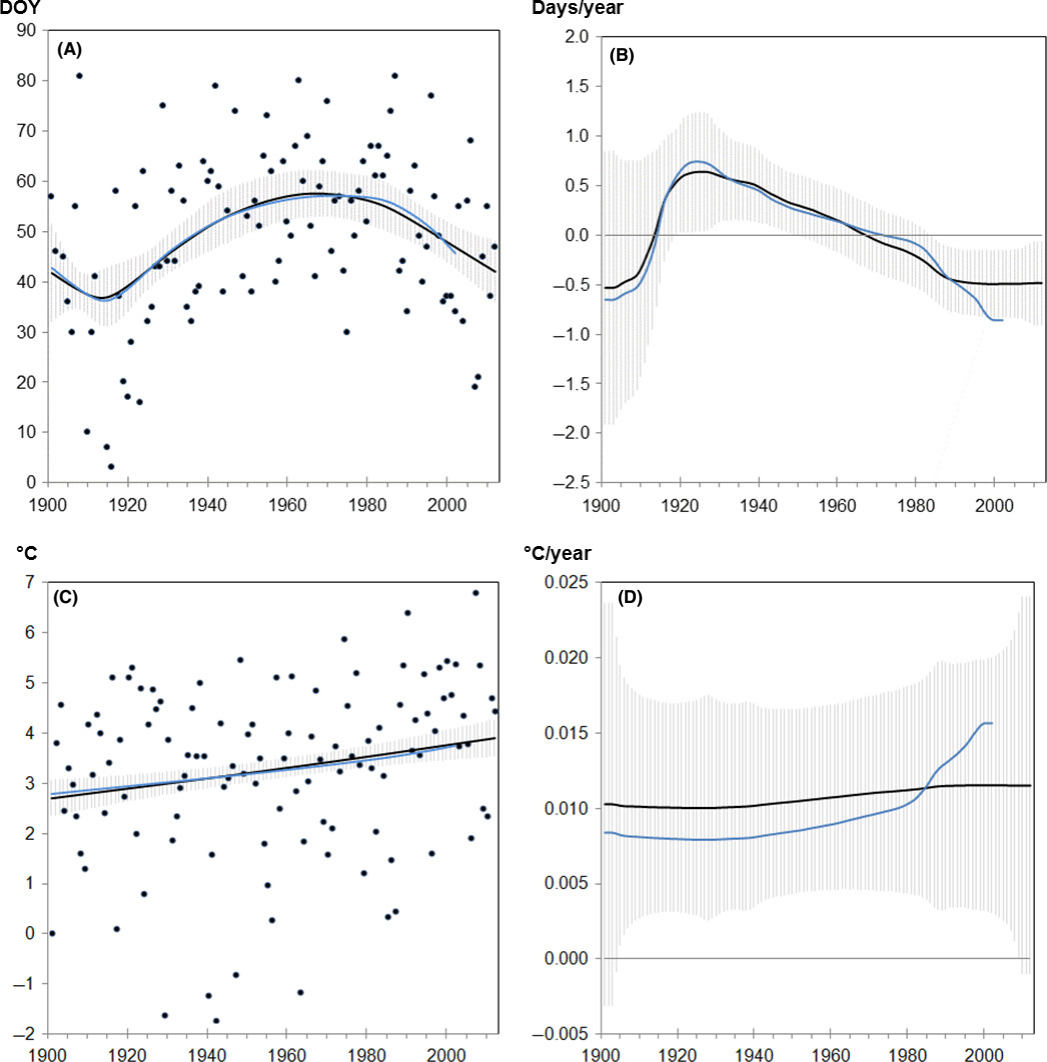
(A, C) Model-averaged functions: Black dots represent observed (A) onset dates [DOY, day of the year] of snowdrop flowering in Geisenheim and (C) temperature means from January to March. Corresponding rates of changes for (B) flowering [days/year] indicating advancements (negative values) or delays (positive values) and (C) temperature [°C/year] indicating increases (positive values) or decreases (negative values; not applicable). Solid lines are the estimates of the fit function/derivative calculated for the dataset ending either in 2002 (blue line) or 2012 (black line), gray shading: confidence intervals (± standard error) around the expectation value.

Figure2A–D display the model-averaged functions and temporal rates of change for snowdrop flowering and temperature data for January to March at Geisenheim. We incorporated data ending in 2002 (blue lines) and 2012 (black lines) in order to compare the differences in functions and trends. The confidence intervals (± standard error) of the 2012 results are indicated by a gray shading; the increase in uncertainty at the beginning and end of the time series and near the change points is induced by a reduced number of data for the estimation of function values. Considering the shorter record (1901–2002), the model fit ended with slightly lower values, that is, with an earlier onset of snowdrop (Fig.2A), which, in turn, resulted in a more pronounced temporal trend for the shorter time span. This is illustrated in Fig.2B showing the rate of phenological change over time [days/year] for the two data series (−0.86 vs. −0.48 days/year in 2002 and 2012, respectively, see also Table4). The two curves start to deviate from each other around 1990 and flatten out at rather different levels.

**Table 4:** Rate of change (days/year) for flowering of snowdrop, cherry, and lime tree at Geisenheim, Rochlitz, and Teterow and for corresponding temperature data (°C/year; T 1_3: temperature mean of January to March, T 2_4: mean of February to April, T 3_5: mean of March to May) for the end years 2002 and 2012, respectively. Δ difference between 2002 and 2012 in days/year (negative differences indicate a slowing down of the advancing trends) and °C/year (positive values indicate a slowing down of the warming trends)

Phenology	Geisenheim			Rochlitz			Teterow		
	Snowdrop	Cherry	Lime tree	Snowdrop	Cherry	Lime tree	Snowdrop	Cherry	Lime tree
2002	−0.86	−0.44	−0.47	−0.91	−0.02	−0.63	−1.36	−0.18	−0.87
2012	−0.48	−0.30	−0.41	−0.14	−0.17	−0.52	−0.62	−0.23	−0.72
Δ	−0.38	−0.14	−0.06	−0.77	0.15	−0.11	−0.74	0.05	−0.15

Temperature	T 1_3	T 2_4	T 3_5	T 1_3	T 2_4	T 3_5	T 1_3	T 2_4	T 3_5
2002	0.016	0.014	0.017	0.018	0.009	0.007	0.025	0.016	0.015
2012	0.012	0.013	0.050	0.006	0.008	0.033	0.008	0.014	0.044
Δ	0.004	0.001	−0.033	0.012	0.001	−0.026	0.017	0.002	−0.029

Whereas the model-averaged functions for the temperature means of January to March do not show distinct differences in 2002 and 2012 (Fig.2C), the temporal trend at the end of the Geisenheim record is slightly higher when calculated with the record ending in 2002 (Fig.2D, 0.016°C/year vs. 0.012°C/year).

Comparing 2002 and 2012 data, it was obvious that both temperature increase and phenological advance were less pronounced in the longer record ending in 2012. A similar pattern to the above described case of snowdrop flowering at Geisenheim was also observed for snowdrop flowering in Rochlitz and Teterow and for cherry flowering in Geisenheim (Table4 and Figs S1A–D and S2A–D in Supporting Information).

**Figure 3:**
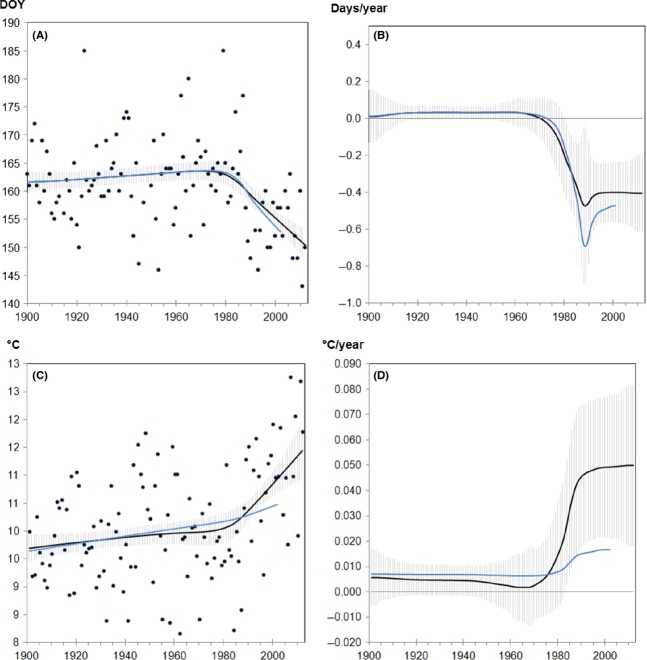
(A, C) Model-averaged functions: Black dots represent observed (A) onset dates [DOY, day of the year] of lime tree flowering in Geisenheim and (C) temperature means from March to May. Corresponding rates of changes for (B) flowering [days/year] indicating advancements (negative values) or delays (positive values) and (C) temperature [°C/year] indicating increases (positive values) or decreases (negative values; not applicable). Solid lines are the estimates of the fit function/derivative calculated for the dataset ending either in 2002 (blue line) or 2012 (black line), gray shading: confidence intervals (± standard error) around the expectation value.

Model-averaged function (Fig.3A) and corresponding rates of change (Fig.3B) for lime tree flowering in Geisenheim revealed only minor differences between the two ending dates (2002: −0.47 days/year vs. 2012: −0.41 days/year). However, model fits of mean March-to-May temperature data (Fig.3C) showed strongly increasing values for the recent two decades linked to a more pronounced trend (Fig.3D) in 2012 (0.050°C/year) compared to 2002 (0.017°C/year).

This means that a slightly weakened phenological trend in 2012 was linked to a more pronounced temperature trend. The same pattern could also be observed for flowering of lime tree in Rochlitz and Teterow (Table4 and Figs S3A–D, S4A–D in Supporting Information). Interestingly, March to May mean temperature data in Teterow were fitted best by a two change point model (Table3, Fig. S4C in Supporting Information).

**Figure 4:**
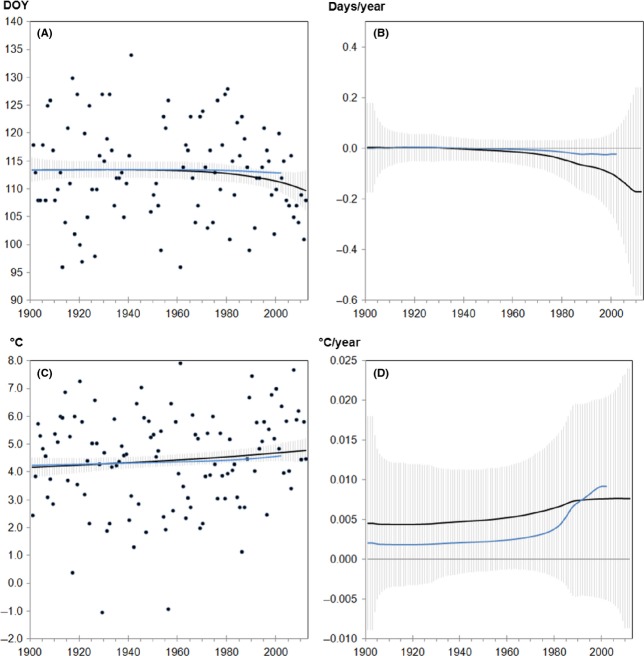
(A, C) Model-averaged functions: Black dots represent observed (A) onset dates [DOY, day of the year] of cherry flowering in Rochlitz and (C) temperature means from February to April. Corresponding rates of changes for (B) flowering [days/year] indicating advancements (negative values) or delays (positive values) and (C) temperature [°C/year] indicating increases (positive values) or decreases (negative values; not applicable). Solid lines are the estimates of the fit function/derivative calculated for the dataset ending either in 2002 (blue line) or 2012 (black line), gray shading: confidence intervals (± standard error) around the expectation value.

The model functions for cherry flowering in Rochlitz confirmed a tendency to earlier onset dates in 2012 compared to 2002 (Fig.4A). The rate of change determined by the multiple change-point model (Fig.4B) was −0.17 days/year in 2012 and was therefore considerably higher than the rate determined by the model for 2002 (−0.02 days/year). Results of the corresponding temperature data, in contrast, were almost identical with respect to the average model function and rates of change (Fig.4C and D).

Thus, cherry flowering in Rochlitz was characterized by a more pronounced phenological trend linked to a less pronounced temperature increase. The same accounted for flowering of cherry in Teterow (Table4 and Fig. S6A–D in Supporting Information).

A summary of all rates of change in recent years is presented in Table4. The trends of blossom onset were smaller in 2012 than in 2002 in seven cases (exceptions: cherry flowering in Rochlitz and Teterow). Rates of temperature change (Table4) were characterized by less pronounced trends in 2012 compared with 2002 for the temperature means of January to March and February to April, but more pronounced trends for the means of March to May, at all analyzed sites.

## Discussion

### One change point vs. multiple change-point model

In contrast to the one change point model with a hockey stick resembling result presented in Dose and Menzel (2004), the multiple change-point approach is characterized by a considerably enhanced structure. This higher flexibility should allow us to describe the temporal behavior of phenology and temperature in long-term records in more detail (see also Pope et al. 2013). Nevertheless, the one change point model (n_p_ = 3) was the favored model for all phenological records and sites analyzed. This means that the results published by Dose and Menzel (2004) are still valid and that the progression of phenological onset dates in Central Europe is predominately characterized by major changes in the early/mid 1980s.

As the one change point model presented in Dose and Menzel (2004) did not account for averaged model fits weighted with their respective probability and was consequently reduced in structure of the model fit, the presented phenological rates of change were overestimated compared with the results based on complete Bayesian reasoning averaging all model types with their respective probability. Dose and Menzel (2004) reported for 2002 at Geisenheim an advance of −1.5 days/year for snowdrop flowering (in this study using the same record: −0.86 days/year) and an advance of −0.6 days/year for cherry and lime tree flowering (in this study: −0.44 and −0.47 days/year).

In most of the cases analyzed, we could demonstrate that the linear model was the dominant model when temperature data were considered. Recent strong increases in March to May temperatures might be responsible for the one (Geisenheim) and two change point model (Rochlitz) being favored which resulted in quite different model fits compared to the mean temperatures of January to March and February to April.

### Rates of change 2002 vs. 2012

The evaluation of variation in phenological and temperature trends for 2012 compared to 2002 showed that there were partially contradictory results for phenological onset dates and temperature means. In four of nine cases, temperature increase and phenological advance were less pronounced in the longer record ending in 2012. This was true for early phases of snowdrop and cherry flowering at Geisenheim and snowdrop flowering at Rochlitz and Teterow. Although recent temperature trends appeared to have become flat (often reported as pausing/standstill in climate change, temporary hiatus in global warming (Hansen et al. 2013; Fyfe et al. 2013), our findings reveal that changes in temperature are still mirrored in changes in phenology in a consistent way. Flowering of snowdrop, the earliest phenophase analyzed in this study, was the only one that universally reflected temperature changes. This might be attributable to the fact that early flowering species better indicate changes in temperature (Menzel et al. 2006) and require less chilling and forcing temperatures (Jeong et al. 2011), making them sensitive indicators of temperature changes.

A less pronounced advancing trend in phenology was linked to an intensifying temperature trend in 2012 for lime tree flowering at all three sites, Geisenheim, Rochlitz and Teterow. More precisely, the temperature warming trends of this later period (March to May) increased from 2002 to 2012 (see Table4). A prominent factor often specified to lead to less pronounced advances in phenology is the lack of winter chilling. As chilling requirements are species-specific (Hänninen and Tanino 2011), our data suggest that snowdrop might be less dependent on low winter temperatures than lime tree. In addition, it has been suggested that under high temperature, the limiting factor in phenological advancement has shifted from temperature to light requirements (i.e., day length, Morin et al. 2010) implying that further temperature increases are not equally matched by corresponding phenological advances.

Only two of nine relationships (cherry flowering in Rochlitz and Teterow) were characterized by a more pronounced rate of phenological change in 2012 than in 2002 connected with a more or less unchanged temperature trend in 2012 compared to 2002. Although trends of February to April temperatures at Rochlitz and Teterow for the shorter and longer period were almost identical (Table4), the observed changes in phenology did not match the observed changes in temperature, but exaggerated them. This might hint to a nonlinear relationship between phenology and temperature, however, there are hardly any factors which could explain this intensification of a phenological response to warming.

To conclude, seven of nine phenological phases indeed slowed down their advancing trends in the last decade. However, early phenological phases, such as snowdrop flowering, seem to be capable of mirroring also temporal variations in trends, whereas for phases in late spring, their power of fingerprinting small variations in temperature increase (e.g., pausing or intensification) seems to be restricted.

There are several aspects discussed for a failure of fingerprinting climate change in phenological data. Besides temperature, several external and internal factors can also influence plant phenology: for example, photoperiod (Leopold 1951), precipitation (Rathcke and Lacey 1985), edaphic factors (Wielgolaski 2001), nutrient availability (Jochner et al. 2013), pollutants (Cape 2003; Honour et al. 2009), genetics (Baumgartner 1952), plant age (Menzel and Fabian 1999; Rosenzweig et al. 2008), or size (Seiwa 1999). In addition, the interactions of meteorological drivers such as temperature, precipitation, and solar radiation on their phenological response are far from yet adequately understood (Morisette et al. 2009). The impacts of extreme events might be more substantial than mere changes in mean temperatures: Heat waves (Jochner et al. 2011) and drought (Jentsch et al. 2007), for example, were found to influence phenological development dramatically. Delayed impacts of environmental factors in onset dates can also be attributed to the annual cycle of trees which is an integrated system with one phenophase affecting the subsequent phases (Hänninen and Tanino 2011).

Although classical statistical methods such as linear trend analyses are suitable to detect changes, especially when temperature is considered, our study demonstrated that the multiple change-point approach is also useful for the description of time series. This was particularly true for phenological data as changes over time were not strictly linear. Analyses conducted with records ending in 2002 demonstrated distinct differences when compared with analyses ending in 2012. As a Bayesian model fit provides the annual rates of change a more detailed understanding of environmental changes can be retrieved. Extrapolation of trends derived from datasets with different lengths could lead to discernible differences and most recent data should be integrated in order not to overestimate or underestimate future phenological changes.

## Supporting Information

**Figure S1.** (A, C) Model-averaged functions: Black dots represent observed (A) onset dates [DOY, day of the year] of snowdrop flowering in Rochlitz and (C) temperature means from January to March. Corresponding rates of changes for (B) flowering [days/year] indicating advancements (negative values) or delays (positive values) and (C) temperature [°C/year] indicating increases (positive values) or decreases (negative values; not applicable).

**Figure S2.** (A, C) Model-averaged functions: Black dots represent observed (A) onset dates [DOY, day of the year] of snowdrop flowering in Teterow and (C) temperature means from January to March. Corresponding rates of changes for (B) flowering [days/year] indicating advancements (negative values) or delays (positive values) and (C) temperature [°C/year] indicating increases (positive values) or decreases (negative values; not applicable).

**Figure S3.** (A, C) Model-averaged functions: Black dots represent observed (A) onset dates [DOY, day of the year] of lime tree flowering in Rochlitz and (C) temperature means from March to May. Corresponding rates of changes for (B) flowering [days/year] indicating advancements (negative values) or delays (positive values) and (C) temperature [°C/year] indicating increases (positive values) or decreases (negative values; not applicable).

**Figure S4.** (A, C) Model-averaged functions: Black dots represent observed (A) onset dates [DOY, day of the year] of lime tree flowering in Teterow and (C) temperature means from March to May. Corresponding rates of changes for (B) flowering [days/year] indicating advancements (negative values) or delays (positive values) and (C) temperature [°C/year] indicating increases (positive values) or decreases (negative values; not applicable).

**Figure S5.** (A, C) Model-averaged functions: Black dots represent observed (A) onset dates [DOY, day of the year] of cherry flowering in Geisenheim and (C) temperature means from February to April. Corresponding rates of changes for (B) flowering [days/year] indicating advancements (negative values) or delays (positive values) and (C) temperature [°C/year] indicating increases (positive values) or decreases (negative values; not applicable).

**Figure S6.** (A, C) Model-averaged functions: Black dots represent observed (A) onset dates [DOY, day of the year] of cherry flowering in Teterow and (C) temperature means from February to April. Corresponding rates of changes for (B) flowering [days/year] indicating advancements (negative values) or delays (positive values) and (C) temperature [°C/year] indicating increases (positive values) or decreases (negative values; not applicable).
