# Supplementary figures and images for: Does flower phenology mirror the slowdown of global warming?
Source: Ecol Evol. 2015 May 15;5(11):2284–95. doi: 10.1002/ece3.1503 (PMC4461427; doi:10.1002/ece3.1503)

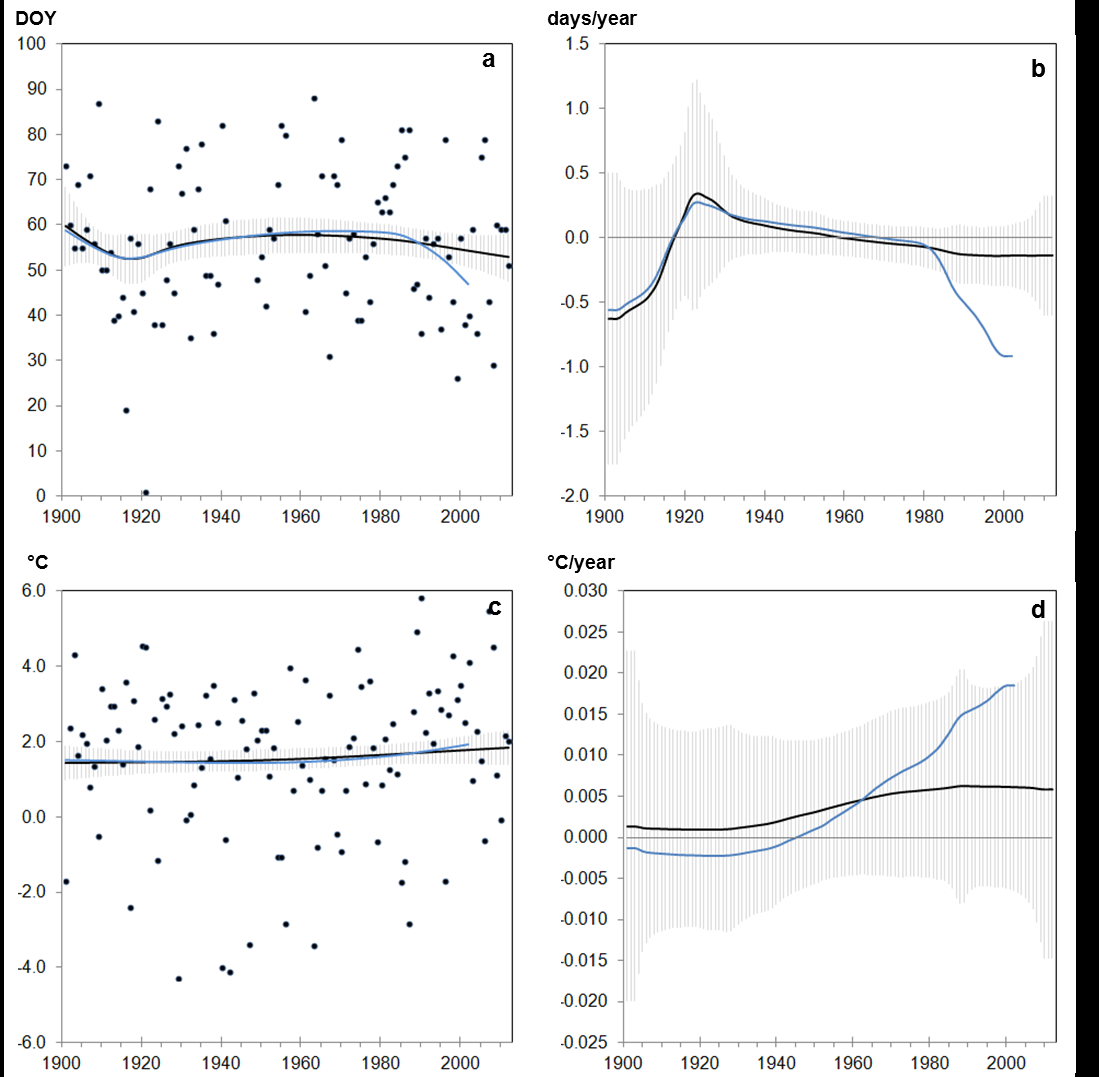

Supplement: Supplementary file 1 [file ece30005-2284-sd1.tif]

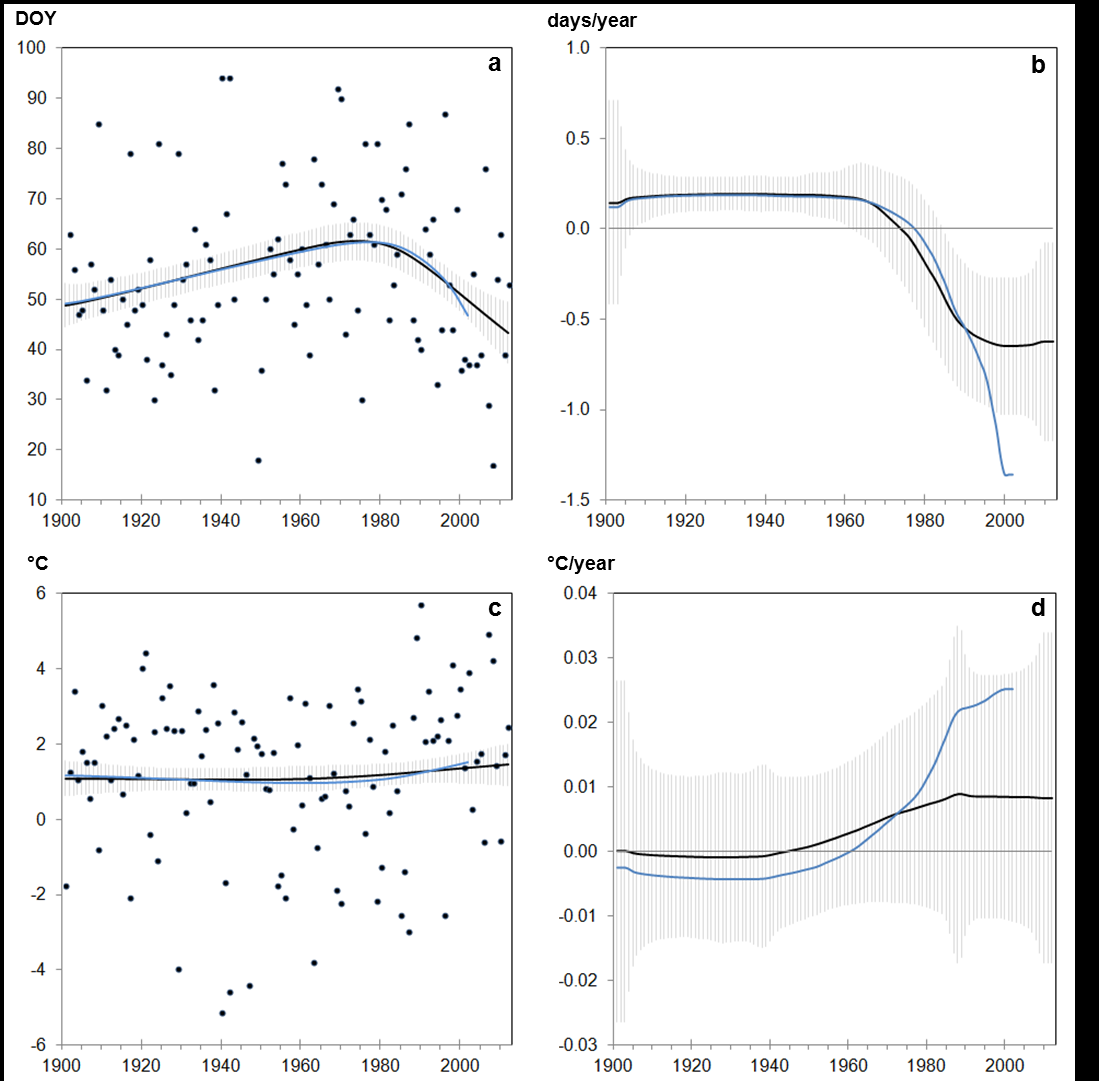

Supplement: Supplementary file 2 [file ece30005-2284-sd2.tif]

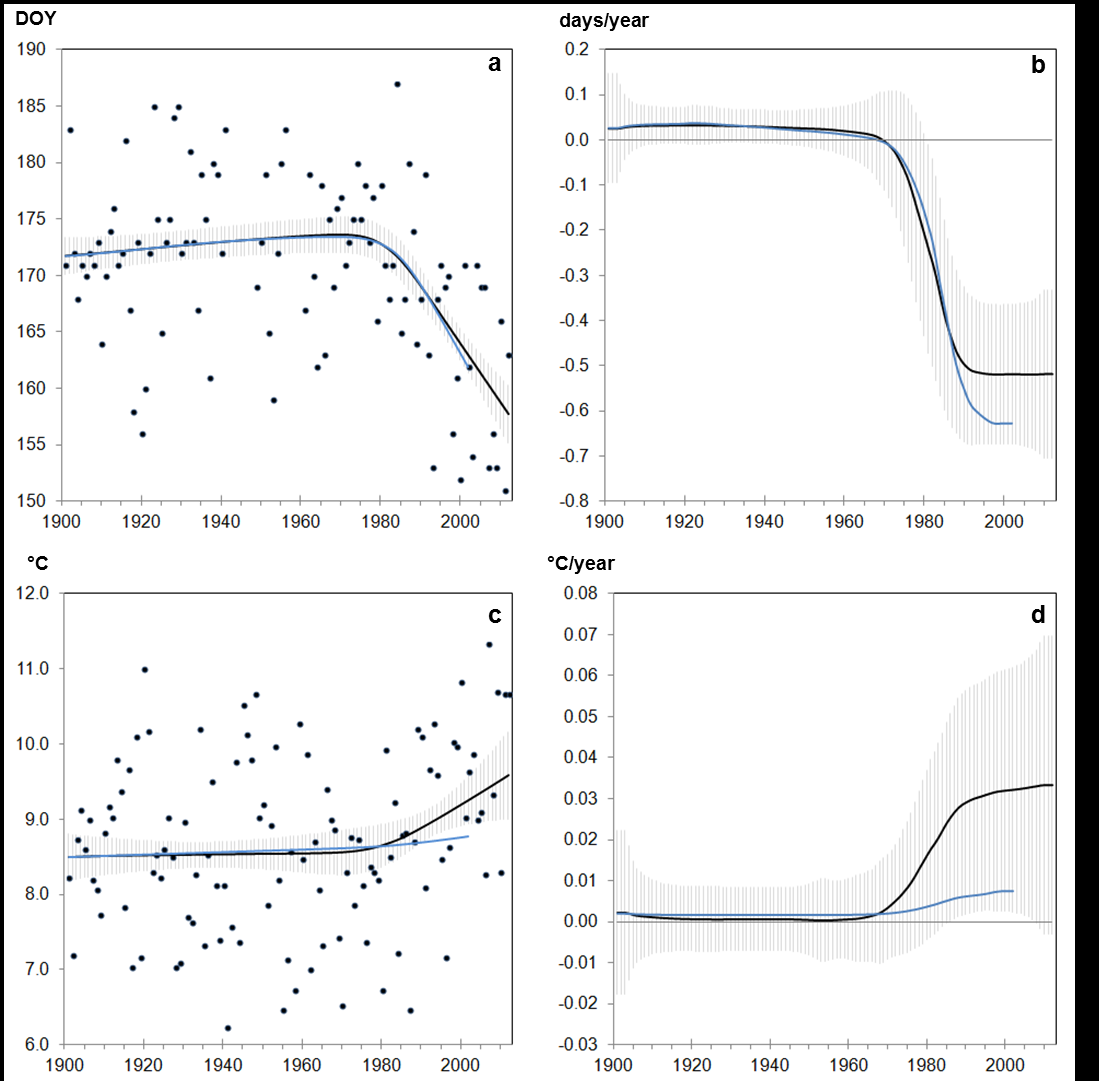

Supplement: Supplementary file 3 [file ece30005-2284-sd3.tif]

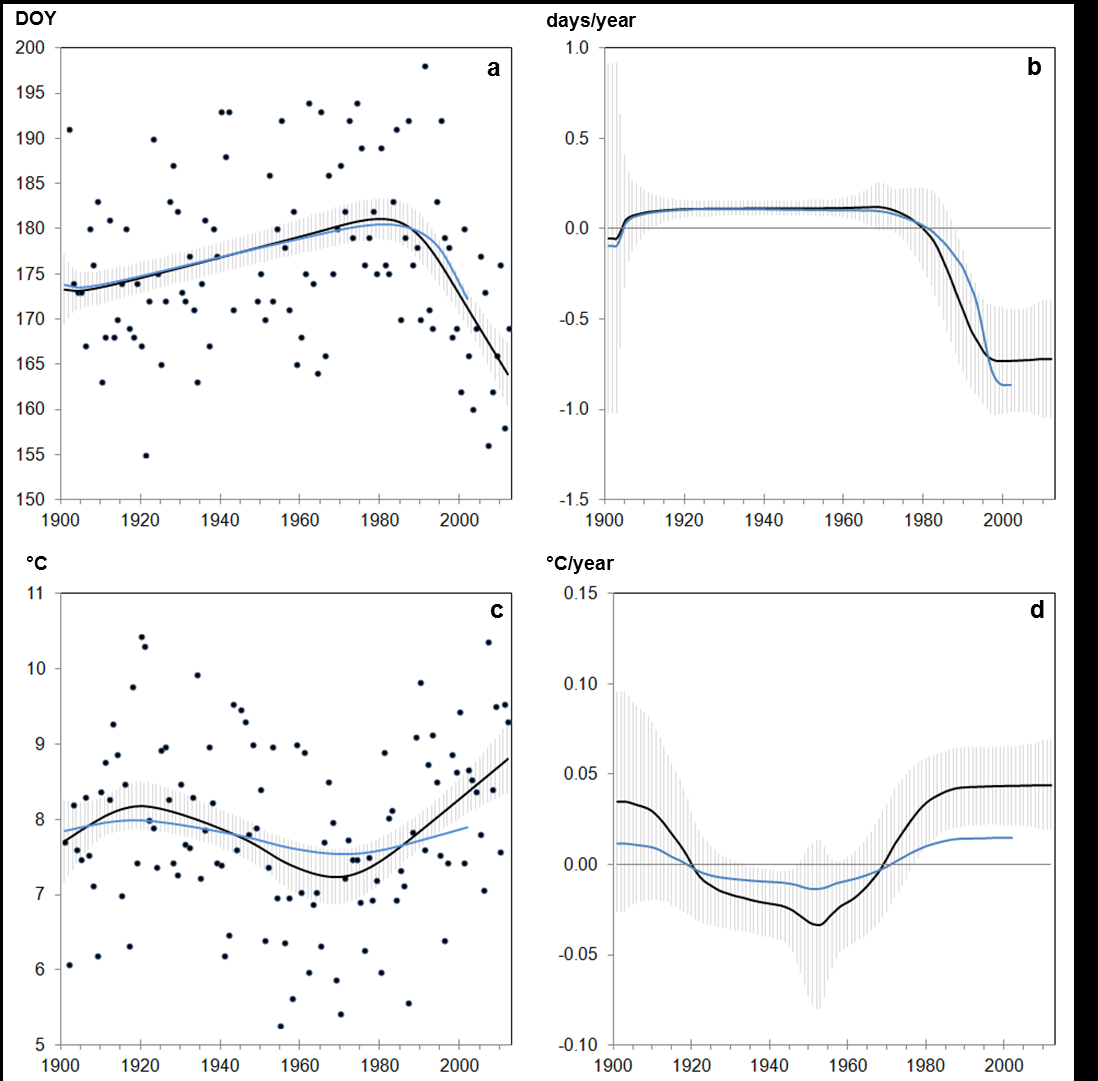

Supplement: Supplementary file 4 [file ece30005-2284-sd4.tif]

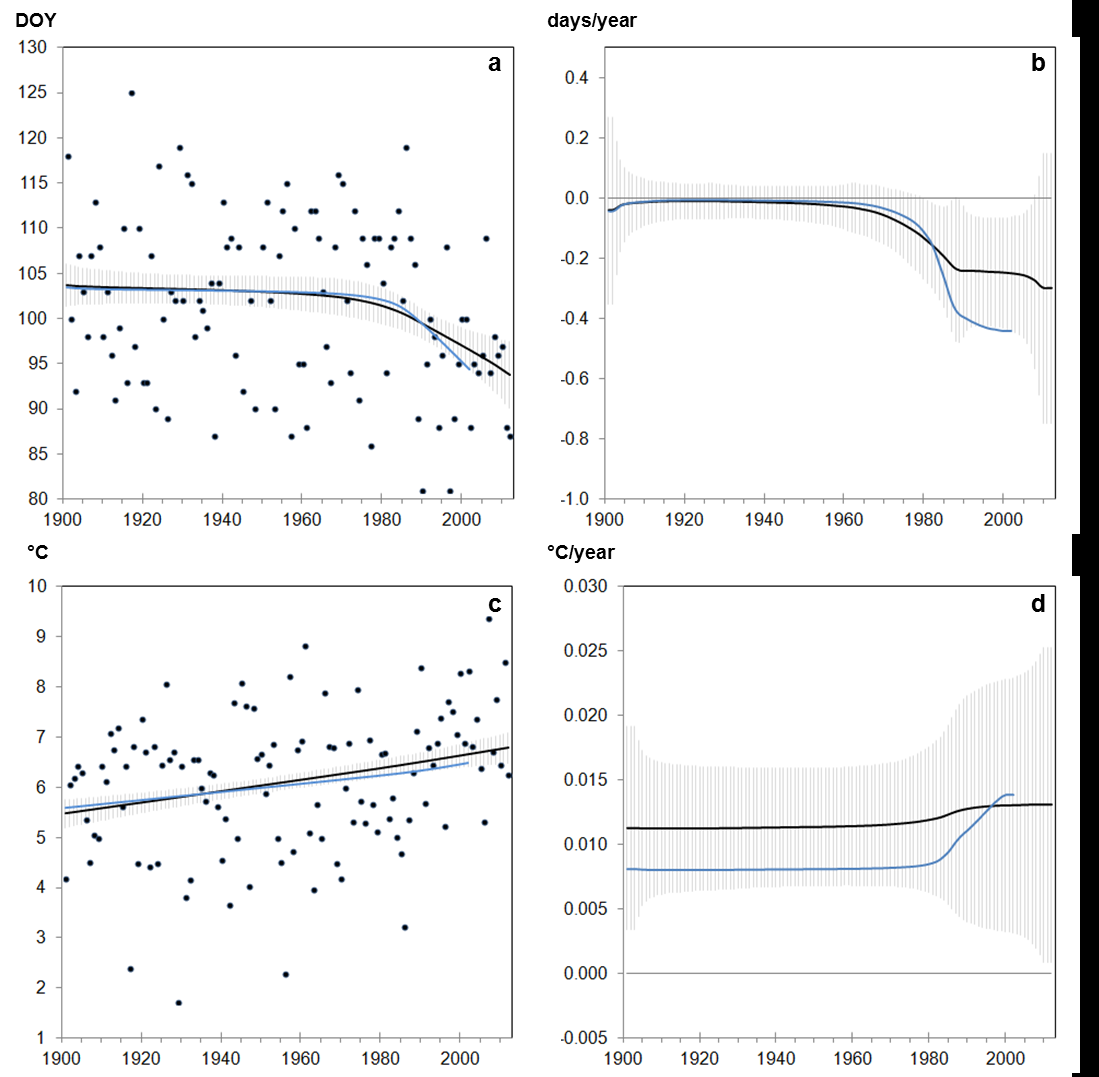

Supplement: Supplementary file 5 [file ece30005-2284-sd5.tif]

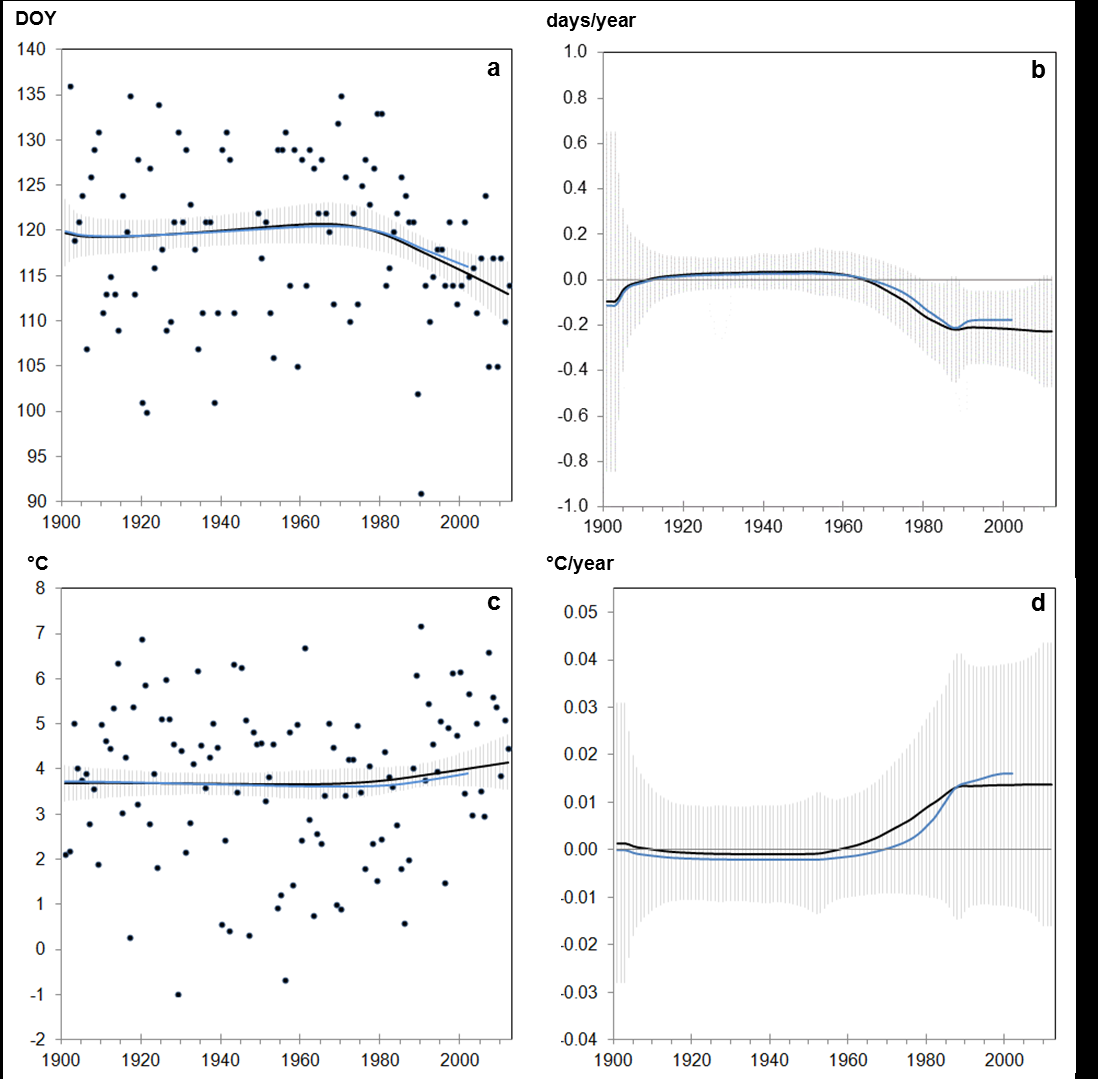

Supplement: Supplementary file 6 [file ece30005-2284-sd6.tif]
